# The role of genomic signatures of directional selection and demographic history in the population structure of a marine teleost with high gene flow

**DOI:** 10.1002/ece3.9602

**Published:** 2022-12-08

**Authors:** Peggy Weist, Sissel Jentoft, Ole K. Tørresen, Franziska M. Schade, Christophe Pampoulie, Uwe Krumme, Reinhold Hanel

**Affiliations:** ^1^ Thünen Institute of Fisheries Ecology Bremerhaven Germany; ^2^ Department of Biosciences, Centre for Ecological and Evolutionary Synthesis University of Oslo Oslo Norway; ^3^ Thünen Institute of Baltic Sea Fisheries Rostock Germany; ^4^ Marine and Freshwater Research Institute Hafnafjörður Iceland

**Keywords:** adaptive variation, candidate gene, divergence hitchhiking, genome assembly, ice ages, single nucleotide polymorphism

## Abstract

Recent studies have uncovered patterns of genomic divergence in marine teleosts where panmixia due to high gene flow has been the general paradigm. These signatures of divergent selection are often impacted by structural variants, acting as “supergenes” facilitating local adaptation. The highly dispersing European plaice (*Pleuronectes platessa*)—in which putative structural variants (i.e., inversions) have been identified—has successfully colonized the brackish water ecosystem of the Baltic Sea. Thus, the species represents an ideal opportunity to investigate how the interplay of gene flow, structural variants, natural selection, past demographic history, and gene flow impacts on population (sub)structuring in marine systems. Here, we report on the generation of an annotated draft plaice genome assembly in combination with population sequencing data—following the salinity gradient from the Baltic Sea into the North Sea together with samples from Icelandic waters—to illuminate genome‐wide patterns of divergence. Neutral markers pointed at large‐scale panmixia across the European continental shelf associated with high gene flow and a common postglacial colonization history of shelf populations. However, based on genome‐wide outlier loci, we uncovered signatures of population substructuring among the European continental shelf populations, i.e., suggesting signs of ongoing selection. Genome‐wide selection analyses (xp‐EHH) and the identification of genes within genomic regions of recent selective sweeps—overlapping with the outlier loci—suggest that these represent the signs of divergent selection. Our findings provide support for genomic divergence driven by local adaptation in the face of high gene flow and elucidate the relative importance of demographic history versus adaptive divergence in shaping the contemporary population genetic structure of a marine teleost. The role of the putative inversion(s) in the substructuring—and potentially ongoing adaptation—was seemingly not substantial.

## Graphical abstract

Marine teleosts are a textbook example of high gene flow species. However, a growing body of literature has identified locally adapted populations though the underlying mechanisms in shaping a population substructure in the face of high gene flow are still not fully understood in these species. We used whole‐genome sequencing information to illuminate the population genetic structure of a marine flatfish with highly dispersive pelagic stages sampled along a steep salinity gradient and to infer the demographic history of populations. While neutral markers revealed genetic homogeneity across large parts of the European continental shelf, genome‐wide distributed outlier loci pointed at a distinct population substructure even at small geographic scales. Our findings suggest genomic divergence facilitated by local adaptation and point to the relative importance of demographic history and adaptive variation in shaping the contemporary population structure.

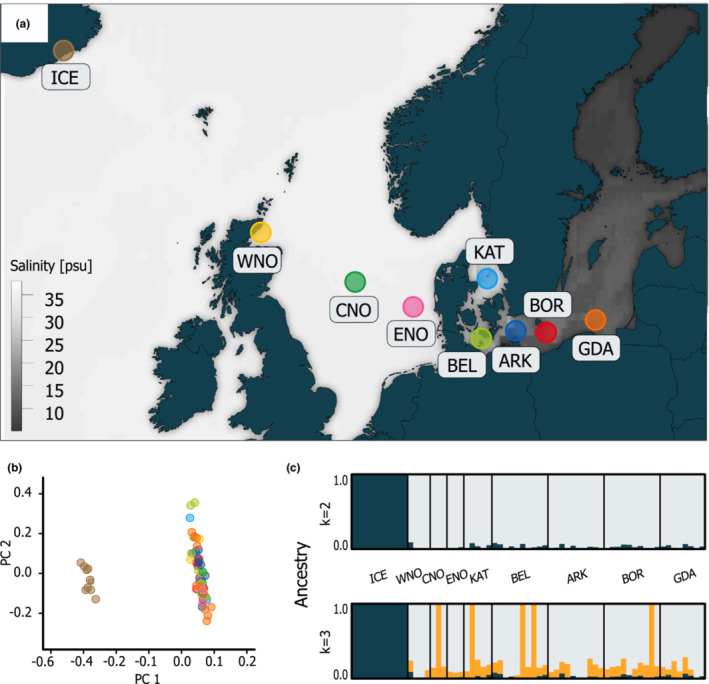

## 1 INTRODUCTION

Recent progress in state‐of‐the‐art genomic tools has been most helpful in our understanding of the evolutionary trajectory of nonmodel organisms in the last decade (Ekblom & Galindo, 2011; Helyar et al., 2011). Such comprehensive characterizations have included genome‐wide patterns of divergence, e.g., loci that are under adaptive selection or genetic drift, as well as neutral genomic variation, which are indicative of demographic processes such as gene flow, migration, and dispersal (Ellegren & Galtier, 2016; Gagnaire et al., 2015). In marine teleosts, the absence of physical barriers to migration and larval dispersal promotes genetic connectivity even between geographically distant populations (Cowen et al., 2000). Genomic signatures of local adaptation are expected to be rare in such species since strong directional selection pressure is needed to override the effect of random genetic drift and of migration between populations (Tigano & Friesen, 2016). However, there is an increasing number of studies demonstrating patterns of genetic and genomic divergence and thus, evidence for signatures of local adaptation in marine teleosts despite high gene flow (Barth et al., 2017; Hemmer‐Hansen et al., 2007). These investigations have largely contributed to our understanding of the molecular basis of adaptation and identified key sources of adaptive evolution (Feder et al., 2012; Wellenreuther et al., 2019). Structural variants—i.e., smaller or larger genomic reorganizations including chromosomal inversions—have been demonstrated as potent forces in driving local adaptation and diversification allowing for the inheritance of co‐adapted gene complexes (Wellenreuther et al., 2019) and play an important role in maintaining genomic regions of divergence even in a milieu of high gene flow (Pettersson et al., 2019). Such chromosomal inversions can act as “supergenes” facilitating local adaptation, by the fact that they encompass multiple neighboring genes with potential beneficial mutations that are inherited together because of close genetic linkage due to lower recombination within these larger haplotype blocks (Tigano & Friesen, 2016). For instance, in Atlantic cod (*Gadus morhua*) four larger chromosomal inversions have been demonstrated to discriminate between populations throughout its geographical distribution, as well as the nonmigratory versus migratory ecotypes (Barth et al., 2017; Berg et al., 2015). Such patterns can promote parts of the genome underlying reproductive isolation or adaptation and prevent them from the homogenizing effects of gene flow leading to so‐called genomic islands of divergence. Such genomic islands could also be facilitated by divergence hitchhiking, i.e., when gene exchange between diverging populations is reduced over large genomic regions of several megabases as an accompanying effect of strong divergent selection on genes involved in local adaptation (Via, 2012), and thus increase the degree of differentiation and eventually lead to reproductive isolation among locally adapted populations (Feder et al., 2012). Linked selection has also recently been shown to form genomic islands of divergence between populations of European sea bass (*Dicentrarchus labrax*) during their past geographic isolation (Duranton et al., 2018).

The North Sea and the Baltic Sea represent a marine and a brackish water ecosystem connected by a relatively steep salinity gradient along the shallow straits of the Danish archipelago. During the late Pleistocene, closed freshwater pools were formed in the southern Baltic region from meltwater as the ice retreated northward. The history of the Baltic Sea started when the preceding freshwater lake opened to the North Sea about 8000 years BP (Björck, 1995). Most of the marine species present today in the Baltic Sea descend from a diverse postglacial flora and fauna established circa 8000–4000 years BP at the time when the Baltic Sea was more saline than today (Winterhalter et al., 1981). Postglacial rebound lowered the connection to the North Sea allowing less saltwater to enter the Littorina Sea, which gradually evolved into the current brackish Baltic Sea. The successive colonization of the Baltic Sea and its characteristics—deeper basins that are connected by shallower sills—has stimulated unique adaptations to life in a stratified brackish environment.

Given its evolutionarily young age, a restricted water exchange with the North Atlantic Ocean, and its predominantly brackish conditions, the Baltic Sea constitutes an ideal study system to investigate patterns of genetic variability and to identify footprints of selection. Over the past thousands of years, marine species colonizing the Baltic Sea were exposed to strong selection pressures imposed by the estuarine conditions such as increasing salinity and decreasing oxygen levels with depth. Many species within the Baltic Sea have evolved locally adapted populations showing increased resilience towards lower salinity, oxygen depletion, and higher water temperatures (Johannesson & André, 2006). Several studies revealed the presence of genetic signatures of directional selection in local Baltic Sea fish populations, which were associated with environmental conditions such as temperature, salinity, or oxygen supply (Guo et al., 2016; Nielsen et al., 2009). For example, in Atlantic cod, genome scans detected outlier polymorphisms strongly associated with adaptation to the local environmental conditions, both within the identified chromosomal inversions and at the genome‐wide level, indicating reproductive isolation between the low‐salinity adapted Baltic cod and the adjacent cod populations (Berg et al., 2015). Among others, candidate genes under selection were functionally related to hydration and the development of oocytes. Indeed, eggs from the eastern Baltic cod stock display remarkable phenotypic differences in neutral egg buoyancy compared with populations inhabiting a more saline environment, likely to compensate for water loss by being less permeable (Petereit et al., 2014).

The European plaice (*Pleuronectes platessa*) is a commercially exploited flatfish species distributed along the Atlantic coasts of Europe including the Baltic Sea (Nielsen, 1986). Both fecundity and dispersal capacities (mostly during the larval phase) are considered to be high (Rijnsdorp, 1991). In the North Sea, spawning occurs off the Scottish coast (De Veen, 1962) and offshore in the deeper, more saline parts of the southern North Sea (Harding et al., 1978). The main spawning season lasts from January to March (Rijnsdorp, 1989). After spawning, the pelagic eggs and larval stages are advected north‐eastwards from the spawning to the nursery grounds in the shallow coastal areas of the Wadden Sea (Cushing, 1990). Juveniles remain here for up to 2 years before they move deeper and recruit to adult feeding grounds. Tagging experiments have further uncovered highly directional seasonal migrations from the winter spawning area to the summer feeding ground and indications of strong spawning site fidelity, indicative of potential subpopulation structuring of plaice in the North Sea area (Hunter et al., 2004). Spawning activities were also reported for the Skagerrak and Kattegat areas (Nielsen et al., 2004), as well as in the Baltic Sea (Nissling et al., 2002). In the Baltic Sea, spawning occurs in the deeper, more saline basins of the western Baltic Sea, the Arkona Sea, and the Bornholm basin from November to March, peaking in the first quarter. In this area, a higher frequency of plaice migrations between the basins, and thus, lower site fidelity is reported (Aro, 1989).

Past genetic studies of plaice showed high levels of gene flow across large geographic distances with a sharp genetic break between the European continental shelf and the off‐shelf population around Iceland (Hoarau et al., 2004; Was et al., 2010). The clear genetic differentiation was attributed to tectonic barriers involving bathymetry and ocean currents (Was et al., 2010). However, while microsatellite analyses revealed genetic homogeneity over much of the European continental shelf, samples from the North Sea, Norway, and the Baltic Sea were weakly distinguishable using mitochondrial DNA (Hoarau et al., 2004; Was et al., 2010). The lack of population structure over substantial parts of the species' distributional range has mainly been attributed to a combination of past colonization events, imperfect spawning site fidelity, and the high dispersal ability of the pelagic early developmental stages (Hoarau et al., 2004; Was et al., 2010). Recent attempts using modern sequencing techniques (ddRAD‐sequencing) uncovered differentiation between plaice from the North Sea and the Baltic Sea (Le Moan et al., 2019, 2021; Ulrich et al., 2013), when mapping their sequencing data towards the chromosome‐anchored genome of the closely related Japanese flounder (*Paralichthys olivaceus*; Shao et al., 2017). This divergence was mainly driven by two putative structural variants (Le Moan et al., 2019, 2021)—on chromosome 19 and chromosome 21—that showed to some degree an allele frequency differentiation following the salinity gradient from the North Sea into the Baltic Sea. Higher overall levels of linkage disequilibrium (LD) and increased genomic divergence (*F*_ST_ and nucleotide diversity *π*) were detected in these regions compared with average genome wide (Le Moan et al., 2021). Additionally, they detected a population genetic differentiation among continental shelf samples driven by isolation by distance (Le Moan et al., 2021).

Given the recent improvements in genomic sequencing techniques and data acquisition as well as in the theoretical frameworks of adaptive evolution in the face of high gene flow, divergent selection and its role in shaping locally adapted populations are and will be continually better understood (Tigano & Friesen, 2016). To improve our understanding of plaice population structure and the impact of gene flow and local adaptation in the Baltic Sea, we here, report on a study that has fully exploited the possibilities of high‐throughput sequencing. An annotated draft genome assembly of European plaice was generated using a combination of short‐and long‐read sequencing technologies, Illumina and PacBio, respectively. Access to a draft genome assembly for the species of interest is crucial to improve mappability, obtain a reliable baseline for variant calling to discover and survey genome‐wide polymorphisms, describe the population genetic structure of a high gene flow species, and illuminate how local adaptation affects genomic differentiation between populations (Ekblom & Galindo, 2011; Wellenreuther et al., 2019). Additionally, we have whole‐genome sequenced (to approx. 8× mean coverage) specimens from locations following the salinity gradient from the hypo‐haline Gdańsk deep (GDA) and Bornholm basin (BOR) in the Baltic Sea towards the Arkona basin (ARK) and the transition zone (Belt Sea [BEL] and Kattegat [KAT]) and further into the North Sea (i.e., the Eastern North Sea [ENO] and the central North Sea [CNO]) together with samples from the Western North Sea (WNO) and Icelandic waters (ICE). With the new resources in place, we first re‐evaluate the hypothesis of genetic panmixia of plaice from the European continental shelf. We then elucidate genome‐wide signatures of divergent selection and recent selective sweeps and identify genes that might be functionally associated with adaptation to different environments. We consider the different environmental conditions experienced by plaice across both, small and large spatial scales. Finally, we confirm the previously detected past demographic history of plaice using Marcovian coalescent analyses of past effective population size and reconstruct the colonization of the European continental shelf and the Baltic Sea based on a high‐resolution genome‐wide SNP set.

## 2 MATERIAL AND METHODS

### 2.1 Sequencing and de novo assembly of a reference genome

To assemble a reference genome of the European plaice, we combined data obtained from long‐and short‐read sequencing from a single female specimen of *Pleuronectes platessa* from the Belt Sea (Table S1) since females are expected to be the heterogametic sex in plaice (Purdom, 1972). High molecular weight DNA was extracted from liver tissue using a high salt precipitation method (https://www.mn.uio.no/cees/english/people/researcher‐postdoc/jentoft/sop‐038‐high‐salt‐dna‐extraction.pdf). DNA was purified using HighPrep polymerase chain reaction (PCR) magnetic beads (MAGBIO). DNA integrity was assessed on a 1%‐agarose gel and DNA content was quantified using a Qubit 3.0 Fluorometer (Invitrogen). Long‐read libraries were prepared using the Pacific Biosciences 20 kb library preparation protocol and sequenced aiming at a coverage of 30× on a Pacific Biosciences RS II instrument. All library preparation and sequencing steps mentioned in this study were performed at the Norwegian Sequencing Centre (NSC), CEES, UiO, Norway. Short‐insert (550 bp) libraries were constructed using the TruSeq DNA PCR‐Free LT Library Preparation Kit (Illumina), and libraries were paired‐end sequenced with read lengths of 250 bp (Rapid Run Mode) aiming at a coverage of 120× on an Illumina HiSeq 2500 system.

The PacBio raw reads were then assembled based on an overlap size set at 2500 bp and initially scaffolded using the software Flye v2.3 (Kolmogorov et al., 2019). The draft genome was further scaffolded using sga v0.10.15 (Simpson & Durbin, 2012) and polished by applying the Arrow algorithm provided within the GenomicConsensus package v2.1.0 (Chin et al., 2013) by mapping back raw PacBio reads onto the preliminary genome. A second polishing step used the short reads generated by Illumina HiSeq 2500 to further improve and error correct the genome assembly as implemented in the software Pilon v1.8 (Walker et al., 2014).

The genome size for the plaice was calculated based on the 17‐mer distribution with Jellyfish v2.1.4 (Marçais & Kingsford, 2011). We assessed the genome completeness by detecting a set of universal single‐copy orthologs (*N* = 4584) specific to Actinopterygii using BUSCO v3.0.1 (Seppey et al., 2019). The repetitive component of the final plaice assembly was estimated using RepeatMasker v4.0.6 (Smit et al., 2015) and the vertebrate database (Bao et al., 2015).

### 2.2 RNA‐sequencing, transcriptome assembly and genome annotation

We isolated RNA from gill, spleen, and liver tissue from a total of 12 adult female plaice individuals caught in the Baltic Sea (Table S1). RNA was extracted using the Total RNA Kit (peqGold), and 150 bp paired‐end libraries were prepared using the TruSeq DNA PCR‐Free Library prep kit for sequencing on an Illumina HiSeq 4000 instrument. Raw reads were adapter trimmed and quality filtered using Trimmomatic v0.36 (Bolger et al., 2014) and assembled using Trinity v3.2.3 (Grabherr et al., 2011) with default parameters.

For gene prediction and annotation, we followed the pipeline implemented in Funannotate v1.5.2 (http://www.github.com/nextgenusfs/funannotate), which combines multiple ab initio gene model predictions with evidence (transcripts or proteins) aligned to the genome and outputs consensus gene models. We used the Trinity assembly as transcript evidence. We further generated a second transcript evidence by mapping the RNA reads to the plaice reference genome with Hisat v2.1.0 (Kim et al., 2015) and assembled the resulting alignments into potential transcripts with Stringtie v1.3.1 (Pertea et al., 2015). Uncertain splice junctions were identified and filtered from the transcripts using Portcullis v1.2.2 (Mapleson et al., 2018). The software Mikado v1.2.4 (Venturini et al., 2018) was used to define expressed loci and a set of “best” gene models. We passed the transcripts to BRAKER v1.9.3 (Hoff et al., 2016), a tool for ab initio gene prediction. Transcript evidence and gene models were then submitted to Funannotate and gene prediction was run with the zebrafish (*Danio rerio*) set as seed species and a maximum intron length of 100 kb. We assessed the completeness of our transcriptome annotation with BUSCO v3.0.1 (Seppey et al., 2019).

### 2.3 Genome sequencing and data analysis

#### 2.3.1 Sampling and sequencing

**FIGURE 1:**
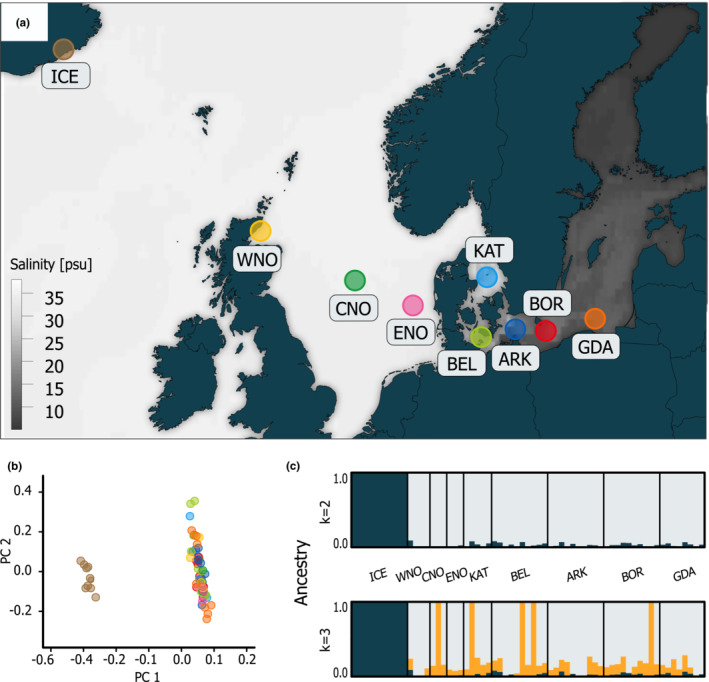
Population structure analysis from a total of 63 European plaice samples based on 83,011 genome‐wide distributed single nucleotide polymorphisms (SNPs). (a) Map of sampling locations of plaice individuals included in this study (see also Table S1). (b) Principal component analysis (PCA) of population differentiation. Individuals are color‐coded corresponding to the sampling locations in (a). (c) Genetic clustering according to an analysis with admixture for *k* = 2 (above; most likely number of putative ancestral populations) and *k* = 3 (below). Genetic clusters are represented by different colors with vertical bars showing the ancestry proportion for each individual genotype.

Tissue from 53 individuals was collected at eight sampling sites covering the species' distribution from Moray Firth in the western North Sea to Gdańsk Bay in the eastern Baltic Sea, representing the edge of the species distribution area (Table 1, Table S1, Figure 1a). Adult mature individuals, either in ripening or spawning conditions were obtained during scientific surveys and from commercial catches. In addition, we included 10 samples from Iceland as outgroup samples, since they are known to be divergent from plaice from the continental shelf (Was et al., 2010). DNA was extracted from each individual using the high salt extraction protocol. Each of these 63 individuals was 150 bp paired‐end sequenced (insert size 350 bp) with the Illumina HiSeq 4000 sequencing technology.

**TABLE 1:** Sample characteristics of plaice individuals (*N* = 63) considered for sequencing and analysis of population structure.

Sample location	Sample code	Region	No individuals	Month	Year
Iceland	ICE	ICE	10	11	2018
Western North Sea	WNO	SHELF, NS	4	2	2016
Central North Sea	CNO	SHELF, NS	3	2	2016
Eastern North Sea	ENO	SHELF, NS	3	2	2016
Kattegat	KAT	SHELF^a^	5	10	2015
Belt Sea	BEL	SHELF, BS	10	2, 3	2016
Arkona basin	ARK	SHELF, BS	10	11, 2, 3	2015, 2016
Bornholm basin	BOR	SHELF, BS	10	2, 3	2016
Gdańsk deep	GDA	SHELF, BS	8	10	2015

*Note*: Sample location and number of individuals are given per location with the respective population affiliation, as well as the sampling month and year (see Table S1 for more details). Sampling locations were grouped according to geography for the demographic inference and the detection of regions under divergence (BS, Baltic Sea; ICE, Iceland; NS, North Sea; SHELF, European continental shelf).

^a^ All individuals originating from the transition area were excluded from comparisons NS versus BS.

#### 2.3.2 Mapping to the reference genome and variant calling

Raw reads were adapter and quality trimmed using Trim Galore v0.3.3 (Krueger, 2015) and mapped against the final plaice genome assembly using the BWA‐MEM algorithm in BWA v0.7.12 (Li, 2013). Duplicates were marked with Picard tools and indels were locally realigned with GATK v3.7 (McKenna et al., 2010). Variants were called using GATK and were initially filtered to include only biallelic SNPs. Snpeff v4.3 (Cingolani et al., 2012) was used to annotate and predict the effects of genetic variants. We applied adjusted hard filters following GATK's recommendations (Van der Auwera et al., 2013): FS > 20, MQRankSum < 12.5, ReadPosRankSum < 2, QD < 5, MQ < 40.

Additionally, we investigated the utility of a high‐quality reference genome for SNP discovery. Here, using the reference genome of the Japanese flounder (*Paralichthys olivaceus*, Shao et al., 2017), the closest relative of *P. platessa* for which a chromosome‐anchored genome was available, to complement the linkage decay analysis at the chromosome level and investigate the heterogenetic differentiation at the putative inversions. We applied exactly the same filtering steps as described below, see Appendix S1 for more detailed information on the methods and an interpretation of the results.

#### 2.3.3 Identifying levels of neutral gene flow

To investigate the population structure of European plaice, variants within spanning deletions were removed using BCFtools v1.1 (Li, 2011). SNPs with a genotype quality score <20, and read depth (DP) <5, or DP >20 were excluded with VCFtools v0.1.13 (Danecek et al., 2011). We pruned SNPs so that no SNP was within 10 bp of another SNP. We excluded SNPs displaying a minor‐allele count <2 across all populations and SNPs deviating from Hardy–Weinberg equilibrium with a *p*‐value < .0001 using Plink v1.9 (Purcell et al., 2007). Furthermore, variants with a minor‐allele frequency <0.03 and more than 20% missing data per site were removed (hereafter “linked dataset”). Linkage disequilibrium (LD) decay was calculated across each of the 20 largest scaffolds. Additionally, LD decay was calculated across each of the 24 Japanese flounder chromosomes by mapping our raw reads directly towards the reference genome of the Japanese flounder (see Appendix S1). Pairwise *r* ^2^ values were calculated between all SNPs per scaffold (and chromosomes) using VCFtools. Decay plots were created per population (ICE, BEL, BOR) by binning the distance between SNPs in increments of 1 kb and averaging the *r* ^2^ values within each bin. SNPs with pairwise *r* ^2^ values >.1 within sliding windows of 50 kb SNPs were omitted with Plink (hereafter “pruned datasets”).

Genetic differentiation between sampling locations was then inspected via PCA as implemented in the Eigensoft software v6.0.1 (Patterson et al., 2006). Ancestry fractions of individual plaice from all sampling locations were inferred based on a maximum likelihood estimation with the software Admixture v1.23 (Alexander et al., 2009) with *k* ranging from 2 to 10, where the most likely number of genetic ancestries was defined based on the lowest cross‐validation error. Pairwise fixation indices (*F*_ST_) were calculated using the package Stampp v1.5.1 (Pembleton et al., 2013) in r v3.6.0 (R Development Core Team, 2017). Bootstrapping was performed to obtain 95% confidence intervals and *p*‐values, which were adjusted for multiple testing by applying sequential Bonferroni correction (Rice, 1989).

#### 2.3.4 Identifying local adaptation and genomic signatures of selection

Locus‐specific *F*_ST_ estimates between sampling locations with a sufficient number of individuals (i.e., ICE, KAT, BEL, ARK, BOR, GDA) were calculated pairwisely on the pruned dataset using VCFtools and loci were screened for highly differentiated SNPs (i.e., above the 99th percentile; *F*_ST_ ≥ 0.4). Based on this set of high *F*_ST_ SNPs we explored the genetic structure by conducting a PCA analysis using Eigensoft.

To detect genomic regions under recent selection and potential candidate genes involved in driving the heterogeneity or local adaptation between the genetic clusters, we conducted genome scans between the Icelandic (ICE) versus all the continental shelf (SHELF) samples and between the North Sea (NS) versus the Baltic Sea (BS) samples (see Table 1). Nucleotide diversity was calculated separately per scaffold in 20 kb sliding windows with 5 kb overlap using VCFtools for ICE, SHELF, NS (i.e., WNO, CNO, and ENO), and BS (BEL, ARK, BOR, and GDA). Allele frequencies per site were calculated with VCFtools. Allele frequency differences (AFD) between ICE/SHELF and NS/BS were computed and for sites with an AFD above the upper 5% percentile, all surrounding genes within 50 kb up‐ and downstream were extracted.

To discover regions with signs of recent positive selection among the populations, we calculated cross‐population extended haplotype homozygosity scores (xp‐EHH) using the R package rehh v3.0.0 (Gautier & Vitalis, 2012). Comparisons were performed between the pooled ICE/SHELF and NS/BS. Therefore, haplotypes were estimated for scaffolds >500 kb using the unpruned dataset with Beagle v4.1 and default settings (Browning & Browning, 2007), and xp‐EHH scores were calculated for window sizes of 50 kb with 10 kb overlap. Since the ancestral allele was unknown, we set “polarized = F.” Significant SNPs for each of the two comparisons were defined by selecting the top 0.05% of log_10_ *p*‐values. Again, we extracted all surrounding genes within 50 kb upstream and downstream. GO terms were annotated by mapping gene names to the UniProtKB database (The UniProt Consortium, 2018). Furthermore, gene functions were categorized into three different categories (cellular components, molecular functions, and biological processes) with PANTHER (Mi et al., 2013) against the human GO database.

#### 2.3.5 Linking signals of local adaptation to potential structural variants

To investigate the existence of the two putative structural variants on chromosome 19 and chromosome 21 and other potential SVs and further infer their impact on genetic divergence and substructuring of plaice in this geographical region, we here mapped our plaice scaffolds towards the reference genome with Minimap2 (Li, 2018) and calculated the linkage decay for these scaffolds. This was done to take full advantage of the higher mappability to our plaice reference genome, resulting in a higher number of SNPs, and as an add‐on to the LD decay analyses and mapping our raw reads directly towards the Japanese flounder reference genome (see description above and Appendix S1). Additionally, we took a closer look at the scaffolds mapping inside or nearby the putative structural variants using VCFtools to calculate the heterozygosity per site over all populations and to perform pairwise calculations of the genetic differentiation along these two chromosomes, comparing ICE versus BOR, ICE versus BEL and BEL versus BOR in sliding windows of 200 kb with steps of 50 kb. The genetic variation between samples was inspected via PCA. These analyses were also conducted for other scaffolds showing the tendency of higher LD—found on other chromosomes.

For two representative scaffolds mapping to the putative structural variant on chromosome 19, individuals were genotyped and assigned to the respective haplogroup based on the PCA clusters (see Mérot, 2020 for concept). Pairwise differentiation (*F*_ST_) and nucleotide diversity (*π*) between individuals representing the noninverted (HOM_R) and the inverted (HOM_I) haplotypes and linkage decay for individuals from the two haplogroups and from heterozygote individuals (HET) along scaffold_302 and scaffold_159 were calculated as described in the previous sections.

#### 2.3.6 Inferring rates of migration and past population history

To understand the direction of neutral gene flow among the plaice sampling locations we estimated relative migration rates between each of the locations with the R package diveRsity v1.9.90 (Keenan et al., 2013) using *G*_ST_ as a measure of genetic distance. We used the pruned set of SNPs and further removed the high *F*_ST_ SNPs potentially under selection leading to a set of 78,508 SNPs. The relative migration rates were calculated on a dataset down‐sampled to five random individuals per location to ensure a balanced sampling scheme.

We inferred past effective population sizes (*N*_e_) separately for ICE and BS using the multiple sequentially Markovian coalescent (MSMC) method as implemented in the software msmc v2.0.0 (Schiffels & Durbin, 2014). We prepared the input data by following the author's recommendations (https://github.com/stschiff/msmc‐tools) with variant sites filtered for a mean depth of 7×. Scaffolds shorter than 500 kb in length were excluded from the analysis. Variant sites falling within repeat regions were masked to include only sites at which the mapping is of high quality.

We performed five MSMC runs with default settings for individuals from ICE or BS, respectively, where each run included three randomly selected individuals. Since the mutation rate is unknown for *P. platessa*, we applied three different mutation rates (low: *μ* = 2 × 10^−9^, medium: *μ* = 1.5 × 10^−8^, high: *μ* = 3.7 × 10^−8^) to cover the known spectrum of mutation rates in marine teleosts (Feng et al., 2017; Liu et al., 2016). The generation time for plaice was set to 3 years (Rijnsdorp, 1989).

## 3 RESULTS

### 3.1 De novo assembly and annotation of a plaice reference genome

Short‐ and long‐read sequencing yielded 73 and 20 Gb of raw data for the reference individual (see Table S1 for detailed sample characteristics), respectively. Reads were assembled into 4512 scaffolds with a total length of 583,260,484 bases. The longest scaffold was 1.4 Mb in size and scaffold N50 was 0.28 Mb (Table S2). Based on 17‐mer analysis, the estimated genome size was 587 Mb (Figure S1). The quality of the genome assembly was confirmed by successfully identifying 94.7% of complete BUSCOs (Table S3). Only 2.5% of the BUSCO genes were not found in the final assembly. Repetitive sequences make up to 8.8% of the plaice genome (Table S4). These are grouped into interspersed repeats (5.5%), small RNA (0.07%), satellites (0.11%), simple repeats (2.71%), and low‐complexity motifs (0.35%).

RNA‐sequencing initially produced 247 Gb of data, which were used to construct a de novo plaice transcriptome. The final transcriptome was 617 Mb in size, contained 800,892 Trinity genes, and had an N50 contig length of 785 bp based on all transcript contigs. Both genome and transcriptome information were integrated for annotation. Finally, a total of 49,037 gene models were annotated. Running BUSCO on the final plaice annotation revealed 88.7% of the searched orthologs being present and complete, of which 84.7% and 4.0% were single‐copy or duplicated orthologs, respectively. About 7.2% of the BUSCO genes were fragmented and 4.1% of the genes were missing in the annotation.

### 3.2 Genome sequencing and variant identification

The whole genomes of 63 plaice individuals were generated to a mean coverage of 8× (± SD 2×), trimmed, and aligned against the plaice reference genome with an average alignment rate of 98% (± SD 2%) with 92% (± SD 3%) of the mapped reads properly paired (Table S5). Variant calling using the GATK pipeline resulted in the discovery of 23,278,376 biallelic Single Nucleotide Polymorphisms (SNPs). These variants contributed to 42,693,312 predicted effects. For intergenic regions, 10,022,005 predicted effects (23.47%) were identified (Tables S6 and S7). Many SNPs were located upstream (22.81%), or downstream (22.66%) of genes, or were introns (27.18%) (Table S6). Among SNPs located in protein‐coding regions, 808,682 (1.89%) were synonymous variants while 669,227 (1.56%) were nonsynonymous variants (Table S7). Additionally, we used the reference genome of the Japanese flounder to investigate the utility of a cross‐species reference genome for mapping and SNP discovery (Appendix S1, including Table S8 for full details).

### 3.3 Population differentiation of European plaice

In general, we detected a rapidly decreasing linkage decay when analyzing the 20 longest plaice scaffolds for each population (ICE, BEL, BOR) (Figure S2) with LD being higher for BOR than for ICE and BEL.

The population differentiation among all samples of European plaice was investigated (see Table 1, Figure 1a) based on 83,011 genome‐wide distributed pruned SNPs (236,458 SNPs prior to pruning). The level of gene flow was first inferred without prior knowledge of the population structure using a principal component analysis (PCA). Two distinct clusters were observed with the Icelandic samples clearly separated from the continental shelf samples (the first axis explains 2.73% of the observed variation, Figure 1b). The second axis, explaining 2.02% of the variation, revealed high individual variation within clusters and low population structuring among the continental shelf plaice samples, i.e., with no apparent differentiation between the North Sea (i.e., WNO, CNO, ENO) and the Baltic Sea samples (BEL, ARK, BOR, GDA).

We further examined the population genetic structure through admixture analysis with different numbers of potential ancestries ranging from *k* = 2 to 10. We observed a minimum cross‐validation (CV) error at *k* = 2, at which all individuals from Iceland were distinguished from the remaining individuals (Figure 1c). The individuals from the North Sea, Kattegat, and the Baltic Sea were mostly composed of the same ancestry, although some shared a minor ancestral portion with the Icelandic samples. At *k* = 3, we observed a third ancestry component for single individuals from the North Sea, the Kattegat, and the western and the eastern Baltic, although a general pattern was not apparent.

The degree of genetic differentiation measured as *F*_ST_ value was hence highest between the Icelandic samples and all the other samples (*F*_ST_ = 0.02, *p* < .001, Table 2). Comparisons among European continental shelf populations revealed lower *F*_ST_ values and most comparisons were nonsignificant. However, we detected subtle levels of genetic substructure among North Sea samples and between samples from the North Sea and the Baltic Sea (Table 2). Within the Baltic Sea, we observed low levels of genetic differentiation among all four sampling locations.

**TABLE 2:** Estimates of genome‐wide differentiation between samples of European plaice based on 83,011 SNPs

	ICE	WNO	CNO	ENO	KAT	BEL	ARK	BOR	GDA
ICE		<0.001	<0.001	<0.001	<0.001	<0.001	<0.001	<0.001	<0.001
WNO	**0.019**		1	1	1	0.684	1	<0.001	<0.001
CNO	**0.021**	0.002		1	<0.001	1	<0.001	<0.001	<0.001
ENO	**0.015**	−0.003	0.000		1	1	1	1	1
KAT	**0.019**	0.001	**0.003**	0.000		1	1	1	0.072
BEL	**0.019**	0.001	0.001	−0.004	0.000		0.072	0.648	1
ARK	**0.020**	0.001	**0.002**	−0.003	0.001	0.001		0.072	1
BOR	**0.019**	**0.001**	**0.004**	−0.001	0.001	0.001	0.001		1
GDA	**0.018**	**0.002**	**0.005**	−0.001	0.002	0.000	0.000	0.000	

*Note*: Pairwise *F*_ST_ estimates are presented below the diagonal and corresponding *p*‐values above the diagonal. Significant estimates are indicated in bold.

Genome‐wide estimates of the overall nucleotide diversity *π* were extremely low (Table S9). Nucleotide diversity was highest in the Icelandic population and lowest for plaice in the Baltic Sea.

### 3.4 Genomic regions of divergence

Even if the LD measurements are on the lower side, it should be mentioned that we did observe some variation between the scaffolds tested. The complementary LD analysis by mapping the plaice reads directly towards the chromosome‐anchored genome of the Japanese flounder confirmed a similar pattern of linkage decay (Figure S3). However, it should be noted, that even if values are very low, we see a tendency towards a slight increase in LD on chromosome 19 where one of the previous putative SVs for plaice has been detected (Le Moan et al., 2021). Of the two putative SVs reported by Le Moan et al. (2021), we only found evidence for increased LD combined with genomic divergence within a larger genomic region on chromosome 19 (Figure S4), while there was no evidence for higher LD and/or genomic differentiation on chromosome 21 or within the putative structural variant on chromosome 21 (Figure S5). However, we did detect other scaffolds with high genomic divergence coupled to high LD that mapped to chromosomes 4, 5, and 6 on the Japanese flounder genome (see Figures S6–S9a–l).

**FIGURE 2:**
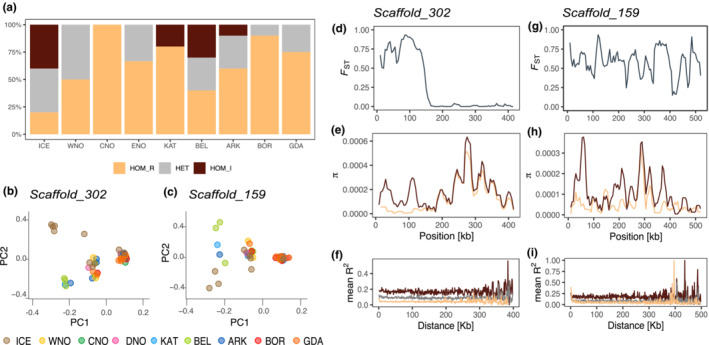
Analysis of linked regions in European plaice for scaffolds mapping to a putative structural variant located in the corresponding chromosome 19 of the Japanese flounder reference genome. (a) Allele frequencies of the putative structural variant based on genotype clustering in (b) and (c) and Figure S11 at different locations following the salinity gradient from the North Sea into the Baltic Sea. The proportion of individuals being homozygous for the presumed collinear allele is shown in yellow (HOM_R), and the proportions of individuals heterozygous or homozygous for the rearranged allele are shown in gray (HET) and dark red (HOM_I), respectively. (b, d–f) Principal component analysis of population differentiation, pairwise differentiation (*F*_ST_), and nucleotide diversity (*π*) between individuals representing the noninverted (HOM_R) and the inverted (HOM_I) haplotypes and linkage decay for individuals from the two haplogroups and from heterozygote individuals (HET) along scaffold_302 and scaffold_159 (c, g–i).

The pattern of population separation seen for this region is showing subtle allele frequency variation from the Baltic Sea into the North Sea (Figure 2a). It should, however, be noted, that within the putative SV on chromosome 19 (SV19) where we localized scaffolds/regions with a higher LD and higher degree of differentiation (Figures 2b–i), we also detected interspersed scaffolds/regions displaying low LD and low differentiation (Figures S4, S10 and S11a–h). Additionally, plaice scaffolds mapping downstream of the structural variant, all demonstrated low LD and no clear genomic separation between locations and/or populations (see Figure S11i–l).

**FIGURE 3:**
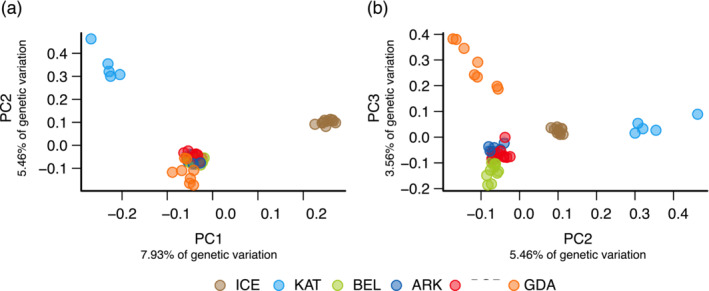
Analysis of 4503 highly differentiated SNPs (*F*_ST_ ≥ 0.4) based on principal component analysis (PCA) of 53 plaice samples for (a) PC1, PC2 and (b) PC2, PC3.

In addition to the allele frequency differentiation along the environmental gradient seen for the potential inversion on chromosome 19, we observed distinct genomic loci of greater differentiation. Notably, 4503 singular loci out of 83,011 pruned SNPs were spotted across all scaffolds with pronounced levels of pairwise differentiation (*F*_ST_ values ≥ 0.4; Table S10, Figure S12). These outlier loci were also distributed all over the genome when scaffolds were mapped against the Japanese flounder genome (figure not shown). Only considering these high *F*_ST_ outlier SNPs, PCA analysis revealed a population substructure that was mostly in congruence with the geographic origin of the individuals (Figure 3a). Considering PC1 versus PC2, the Icelandic and the Kattegat plaice formed isolated clusters, whereas the Baltic Sea samples were not distinguishable. Considering, PC2 versus PC3, samples from the eastern Baltic Sea (GDA) separated from the Baltic Sea cluster (Figure 3b).

Moreover, analyses uncovering genome‐wide signatures of divergent selection and recent selective sweeps and thus, identification of genes that could be functionally associated with adaptation to the different environments of the North Sea and the Baltic Sea were conducted. Here, different environmental conditions experienced by plaice across both, small and large spatial scales were considered. We detected 486 and 358 distinct loci with increased shifts in allele frequency between samples from Icelandic (ICE) and the European continental shelf (SHELF) and from the North Sea (NS) and the Baltic Sea (BS), respectively. Within a distance of 50 kb surrounding the focal SNP in both directions, 167 and 116 annotated genes were identified, respectively (Table S11).

**FIGURE 4:**
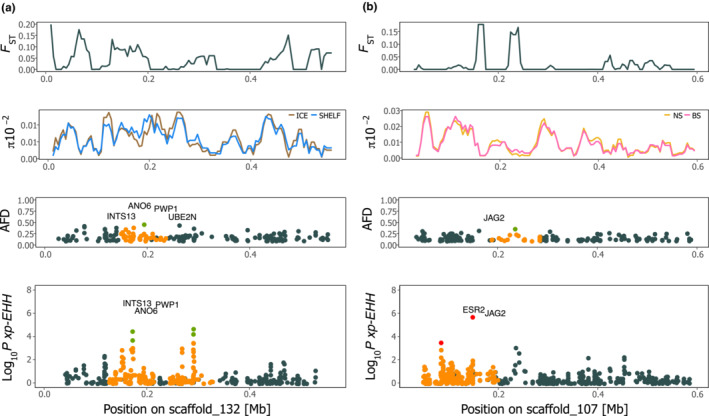
Closer investigation of the genomic differentiation. (a) Genomic region of divergence between individuals from ICE and SHELF (see Table 1) for scaffold_132. Pairwise fixation index (*F*_ST_) between samples and nucleotide diversity (*π*) calculated in sliding windows of 20 kb with 5 kb overlap size. Allele frequency difference (AFD) and cross‐population extended haplotype homozygosity (xp‐EHH) were calculated between samples. Outlier loci above the 99.95th percentile are colored in green, if substitution is synonymous, or red, if substitution is nonsynonymous. Loci within 50 kb distance to the outlier SNP are denoted in orange. (b) Genomic differentiation between NS and BS for scaffold_107.

Genomic regions of divergence were further investigated by identifying signatures of recent selective sweeps. We calculated the cross‐population extended haplotype homozygosity (xp‐EHH) statistic, which compares the integrated extended haplotype homozygosity profiles between two populations at the same locus and points to alleles that have swept to near‐fixation within one of the two populations (Sabeti et al., 2007). Haplotypes were inferred only for the 184 largest scaffolds (i.e., >500 kb), representing 22% of the genome. A total of 232 genomic loci on 44 scaffolds were noted as outliers between ICE versus SHELF and 235 loci on 67 scaffolds for NS versus BS (Table S11, Figure S13). Of these, 54 and 55 SNPs were located in annotated genes, respectively. For ICE versus SHELF, 112 outlier SNPs were located in exons and 20 SNPs were nonsynonymous coding substitutions (Table S11, Figure 4a). For NS versus BS, 110 SNPs were located in exon regions and 36 SNPs were nonsynonymous coding substitutions (Figure 4b). Gene ontology (GO) analysis revealed that many of the genes identified were functionally involved in cellular, metabolic or regulatory processes (Table S11, Figure S14). In terms of biological processes, genes were related to adaptation to low salinity (e.g., PKN1, PKD2), reproduction (e.g., ANTXR1, JAG2), and immune system response (e.g., GPER2, POLR3E).

### 3.5 Direction of gene flow and demographic history

Elucidating directional patterns of genetic connectivity between all pairs of samples from each location revealed moderate to high relative migration rates across the European continental shelf (Figure S15a) supporting the findings regarding the similarity of populations from the PCA analysis (Figure 1b). Highest exchange rates were observed among the Baltic Sea populations. Removing the Icelandic samples revealed a similar pattern among the North Sea and the Baltic Sea populations (Figure S15b). Again, the highest rates of gene flow could be observed within the Baltic Sea.

**FIGURE 5:**
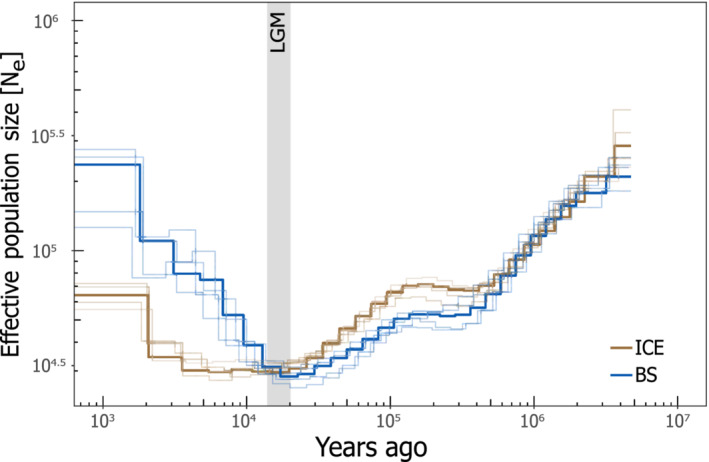
Inference of effective population size (*N*_e_) over time. Past population history was inferred based on five independent runs of MSMC2 for three randomly chosen individuals from the Icelandic (ICE) population and the Baltic (BS) population (Table 1). Results are shown for a medium mutation rate of *μ* = 1.5 × 10^−8^ mutations/site/generation and a generation time of 3 years. The gray bar visualizes the duration of the last glacial maximum (LGM).

Estimates of historical population size (*N*_e_) and demographic trends were inferred using MSMC2 analyses for three different mutation rates and a fixed generation time of 3 years (Figure 5, Figures S16 and S17). After experiencing sharp reductions in *N*_e_ during the Late Pleistocene, both populations gradually expanded in size towards recent times, with the Icelandic populations showing lower levels of *N*_e_ compared with the ancestral Baltic population since the Last Glacial Maximum (LGM). Replicate runs with three randomly chosen individuals yielded consistent *N*_e_ estimates for the Icelandic and the inner Baltic Sea past history. With an assumed mutation rate of 1.5 × 10^−8^ mutations/site/generation, *N*_e_ fluctuated between ~300,000 and ~100,000 for the ancestral Baltic Sea and between ~400,000 and ~50,000 for the Icelandic plaice, respectively.

## 4 DISCUSSION

By generating the first annotated reference genome assembly for the European plaice combined with whole‐genome sequencing of specimens (to approx. 8× coverage) across large parts of its geographical distribution, we uncovered a population substructure that was linked to genomic signatures of local adaptation despite high gene flow within the European continental shelf. This population substructure at smaller geographical scales was only revealed with SNPs potentially under strong selection, i.e., defined as high *F*_ST_ outliers. Neutral markers, however, pointed at the clear separation of the Icelandic population from samples collected along the gradient of the European continental shelf as earlier studies have already revealed. Moreover, by using the whole‐genome information we were able to unfold the demographic history of plaice and detected that the plaice populations investigated experienced a major population bottleneck during the LGM following similar past demographic trajectories.

### 4.1 Genome assembly of the European plaice

We obtained a plaice draft genome assembly of high completeness, covering 99% of the k‐mer‐based estimated genome size. The size of plaice genome assembly (583 Mb) falls within the expected range of flatfish genomes (Chen et al., 2014; Figueras et al., 2016; Shao et al., 2017). However, the estimated genome size is smaller than the genome size calculated with fluorometric methods for Pleuronectidae (Hinegardner & Rosen, 1972). Genomic completeness was further estimated by gene content and gave a high percentage (94.7%) of core gene presence. Compared with other flatfish assemblies the repetitive content in plaice is slightly higher (Chen et al., 2014; Figueras et al., 2016). Along with the repeat content, the level of heterozygosity and low‐complexity regions are known factors impeding the contiguity of a genome assembly (Alkan et al., 2011). Future studies could thus aim for anchoring the 4512 scaffolds that we have detected into the expected number of 48 chromosomes (2n) revealed by the karyotypic analysis in this species (Fan & Fox, 1991) to gain further insight into the genomic architecture of plaice.

Annotation with the Funannotate pipeline initially yielded 49,037 gene models. This is comparatively more than for other flatfish genomes (*Cynoglossus semilaevis*: 21,516 [Chen et al., 2014]; *Paralichthys olivaceus*: 21,787 [Shao et al., 2017]; *Scophthalmus maximus*: 22,751 [Figueras et al., 2016]) for whose annotation different pipelines have been applied. Since the draft assembly is fragmented, it is likely that some of the genes are broken up among several scaffolds leading to an inflation of the overall gene count if some genes are located on different scaffolds (Salzberg, 2019).

### 4.2 Extensive gene flow across the continental shelf

Using whole‐genome sequencing data based on the neutral loci we confirmed the already documented genetic/genomic divergence of the Icelandic and the European continental shelf plaice population and the large‐scale genetic homogeneity across the shelf area (Le Moan et al., 2021; Was et al., 2010). Bathymetry and hydrography have been suggested to act as the main drivers in the formation of the two genetic clusters promoting the reproductive separation of shelf and off‐shelf populations (Hoarau et al., 2004; Was et al., 2010). Moreover, our neutral SNP set uncovered high levels of gene flow between specimens sampled at the North Sea versus the Baltic Sea spawning grounds. However, based on pairwise *F*_ST_ analyses on the pruned/neutral marker set subtle differentiation between plaice from CNO, WNO versus the eastern Baltic Sea samples (ARK, BOR, GDA) was detected (see Table 2). Though, it should be noted that the low number of samples (3–10 individuals per sampling location) used in this study could potentially bias the allele frequency distributions. However, since our results are in line with the previous findings (Hoarau et al., 2004; Was et al., 2010), we propose that several mechanisms including (i) historical events and (ii) selection are important drivers of the contemporary genetic structure of European plaice at different geographical scales.

Congruently, Le Moan et al. (2021) and our study observed the greatest genetic differentiation between plaice from Iceland and the European continental shelf using modern sequencing techniques. Additionally, Le Moan et al. (2021), discovered two putative SVs displaying allele frequency differentiation following the salinity gradient from the North Sea into the Baltic Sea. With the generation of a draft reference genome assembly combined with whole‐genome sequencing data—using a smaller sample set compared with Le Moan et al. (2021)—our results support the existence of a larger genomic region with higher LD on chromosome 19, which shows a pattern of genetic divergence resembling an inversion (see Figure 2b–i, Figure S11a–d), displaying three distinct clusters for the homokaryotypes, the heterokaryotypes, and the homokaryotypes of the alternative arrangement (Mérot, 2020). Moreover, the fact that high LD scaffolds within the putative SV19 were interspersed with regions of low LD and low genetic differentiation (see Figures S4 and S11) could be caused by (i) discrepancy at the micro‐synteny level between the Japanese flounder and the European plaice on chromosome 19, leading to this mosaic pattern of high and low LD regions when mapping towards the Japanese flounder genome, or it could be indicative of (ii) misassembled genomic regions in plaice or (iii) the existence of multiple smaller regions of repressed recombination rather than a single huge block (Pettersson et al., 2019). Alternatively, the alternating pattern of regions with high and low LD is potentially caused by double crossovers (Kennington & Hoffmann, 2013), which have been observed for other species (Kennington & Hoffmann, 2013; Pettersson et al., 2019). Nevertheless, the potential inversion on chromosome 19 is seemingly under balancing selection since all karyotypes were evident at all locations (and subpopulations)—with subtle allele frequency differences—indicative of maintaining all variants within the populations investigated in this study. We further identified additional larger haplotype blocks displaying genomic divergence on chromosomes 4, 5, and 6 pointing at further linked genomic regions that are under selection and thus important for local adaptation. However, a chromosome‐anchored genome of the European plaice could help to improve our understanding of the genomic architecture and patterns of synteny in the teleost family Pleuronectidae.

For many marine fish species, genetic population differentiation is related to their egg and larval dispersal potential, population history, and adult migration (Waples, 1998). Hydrodynamic processes and ocean currents play a major role in linking geographically distant populations by facilitating dispersal during early life stages (White et al., 2010). High fecundity, a long pelagic larval duration, and larval behavior offer great dispersal capacities over hundreds of kilometers by passive transport in plaice (Volckaert, 2013) and by such leading to a high degree of gene flow—as demonstrated by our neutral SNP set—between locations within the European continental shelf at various spatial scales. For instance, in the macrotidal North Sea, currents are important for the dispersion of plaice eggs and larvae from the commonly used spawning grounds in the southern North Sea towards the nursery areas following the direction of the general circulation pattern (Cushing, 1990; Erftemeijer et al., 2009; Hunter et al., 2004) and, thus, enable mixing and genetic homogeneity. In the Skagerrak/Kattegat area larval stages from the North Sea populations passively drifted by currents are retained and thought to return to the North Sea to spawn (Ulrich et al., 2013, 2017), hinting at possible homing behavior in this species. However, the Kattegat samples were genetically similar to the Eastern North Sea and the Baltic Sea samples pointing at imperfect homing and/or the existence of migrants between these sites, respectively. In the Baltic Sea, the exchange of eggs between adjacent basins is suggested to be minor given the species' egg density and that floating eggs are restricted to the more saline bottom layers in each basin (Petereit et al., 2014), potentially resulting in reduced gene flow between spawning grounds in neighboring basins. However, regular major Baltic inflows and periods of strong easterly winds (Mohrholz, 2018) may cause pulsed mixing of eggs and larvae across the sill‐connected adjacent basins of the western and the eastern Baltic Sea. On the other hand, subtle differentiation was noticed between the westernmost North Sea (WNO) and the easternmost Baltic Sea sampling sites (GDA). The subtle pattern might indicate reduced gene flow impacted by multiple evolutionary mechanisms such as isolation‐by‐distance, different selection pressures, and colonization history.

### 4.3 Signatures of local adaptation and putative population substructuring

Although no significant population structure was identified among plaice specimens caught throughout the steep salinity gradient from the Baltic Sea following the shallow straits of the archipelago into the North Sea area based on neutral SNP markers, different results were obtained with highly differentiated SNPs. The indication of a potential population substructuring, i.e., genetic divergence based on the high *F*_ST_ outliers might be explained by strong divergent selection pressures at the various locations and thus point to signatures of local adaptation.

For the North Sea samples, pairwise comparisons of genomic differentiation were omitted due to the limited resolution of allele frequency estimates caused by low sample sizes. Given the subtle signs of genomic differentiation detected among these populations, future studies including more samples from these locations could aim at elucidating the genomic divergence of these populations. Further, the remaining samples from the continental shelf form three genetic clusters (KAT, BEL/ARK/BOR, GDA) based on the high *F*_ST_ outliers roughly corresponding to their geographic origin along the environmental gradient between the North Sea and Baltic Sea. The observed population substructuring within the Baltic Sea area highlights that even within smaller spatial scales, the plaice encounters distinct selection pressures and thus, likely mirrors local adaptations to life in the brackish water environment with deep basins. However, the differentiated GDA might also reflect the effects of living on the edge of the species' distributional range and genetic drift (Johannesson & André, 2006).

Throughout its distribution range, plaice experiences diverse physical environments and variable abiotic conditions, such as salinity, temperature, oxygen content, and tidal currents (in the North Sea), which can ultimately lead to adaptive genomic divergence between locations. Apart from single highly differentiated loci, several genomic regions were strongly differentiated among specimens from the North Sea (WNO, CNO, ENO) and the Baltic Sea (BEL, ARK, BOR, GDA). We detected loci that stood out above the 99.95th percentile in the global comparisons considering either AFD or xp‐EHH (Figure S13) indicating recent selective sweeps. Many of them contained at least one candidate gene with a functional connection to either reproduction, immune system, or adaptation to environmental conditions. For example, on scaffold_132 we detected a region possibly under divergent selection between the continental shelf and off‐shelf populations containing ANO6 (Figure 4a). The gene encodes a transmembrane protein that has been shown to be involved in angiogenesis in zebrafish, a process that is triggered by low‐oxygen conditions (Delcourt et al., 2015). Tolerance against variation in oxygen levels and hypoxia might play an important role in ecological adaptation in plaice and in other demersal species, as well (Barrio et al., 2016), particularly for those living in the Baltic Sea.

On scaffold_107 a nonsynonymous substitution was located in the estrogen receptor ESR2 (Figure 4b), which is suggested to play a vital role during maturation (Vidal et al., 2000). Estrogen receptors have been proposed as candidate genes of selection in Atlantic cod (Clucas et al., 2019) and in three‐spined stickleback (*Gasterosteus aculeatus*; Shimada et al., 2010). In close proximity to ESR2, we identified JAG2, which is involved in spermatogenesis (Hayashi et al., 2001), indicating that the entire genomic region is under strong directional selection. Additionally, we detected multiple genes throughout the genome related to sperm motility (e.g., GAPDH2, INTS13, DDX6), and some were already suggested to be of importance for efficient reproduction in brackish water environments (Berg et al., 2015). Furthermore, some of the genomic regions of divergence were coupled to specific genes important for immunosystem functioning and signaling pathways (e.g., OPRK1 on scaffold_13, DENND1B on scaffold_140).

Our approach to identify outlier loci was rather conservative, i.e., based on the empirical distribution of allele frequencies and haplotype statistics, but a commonly used approach to detect loci that are divergent among populations (Cuevas et al., 2021; Kusakabe et al., 2017; Ravinet et al., 2016). Selection scans such as those applied in this study have the highest power when selection is strong and the genetic architecture underlying a trait under selection is simple (Stephan, 2016). We further note the limited number of samples used for our divergence analysis and caution is still warranted when interpreting the data, but our study takes advantage of genome‐wide variation providing great power in resolving signatures of adaptation.

### 4.4 Reconstruction of plaice population history

Glaciations during the Pleistocene have shaped the contemporary geographical distribution of populations and the degree of genetic connectivity among them (Jenkins et al., 2018). To further illuminate the origin of the population substructure indicated by the outlier SNPs, we reconstructed the demographic history of plaice from individuals from ICE and BS using Marcovian coalescent analyses since high gene flow could have masked past history. It should be noted that the algorithm used here, has some limitations when it comes to low‐coverage data (e.g., as in our dataset). The method has been shown to recover essential features of a demographic history even with lower coverage datasets, however, the estimates of the effective population size might not be accurate (Mather et al., 2020). Assuming a generation time of 3 years and a medium mutation rate of 1.5 × 10^−8^ mutations/site/generation, the results indicate that both populations (ICE vs. BS) have already split more than 200,000 years ago during the mid‐Pleistocene, hinting at two glacial refugia, which has also been suggested by Le Moan et al. (2021). The ancestral population of the Icelandic plaice was probably located close to the Rockall Plateau as hypothesized for Atlantic cod (Pampoulie et al., 2008). The ancestral population of the Baltic plaice likely survived in a glacial refugium located in the northern parts of the North Sea (Kettle et al., 2010) and gradually expanded southwards following the retreat of the glacial coverage during the late Weichselian, a process that was reflected by a decreasing genetic diversity towards the Baltic Sea (this study). Additionally, the lack of population differentiation between the North Sea and the Baltic Sea and the low pairwise differences between sampling sites suggests a substantial genetic exchange between the source population and the Baltic Sea colonizers during past colonization events. After successful colonization, the ancestral Baltic Sea population has rapidly expanded and reached high numbers of contemporary effective breeders. A large effective population size is often correlated with a high evolutionary potential to respond to changing environments (Ellegren & Galtier, 2016). However, a genetic separation may stay unnoticed for a long time since differences in allele frequencies will only slowly accumulate and the signal of population genetic differentiation is likely to be obscured at the level of neutral genomic variation (Hauser & Carvalho, 2008; Waples, 1998). Hence, the combination of high gene flow among European continental shelf plaice, high effective population size, and the relatively young age of the Baltic Sea could have prevented plaice populations living in the North Sea and the Baltic Sea from establishing a distinct population structure at the neutral markers.

## 5 CONCLUSION

Here, genomic markers have unfolded the relative importance of demographic history versus adaptive divergence in shaping the population genetic structure of a marine teleost. We report on the emergence of a putative population substructuring in European plaice whose detection is confounded by high levels of gene flow, a common colonization history of European continental shelf populations, and a high effective population size. While neutral markers revealed the reproductively separated Icelandic and European continental shelf population, outlier loci pointed at a local population structure at smaller geographical scales indicating spatially heterogenous selection and not related to the potential inversion on chromosome 19. Our findings suggest the importance of having access to a reference genome and high‐resolution markers with full‐genome coverage to elucidate how the genomic landscape in plaice is shaped by neutral and adaptive genetic variation and to better understand the evolutionary history of populations. Interestingly, this study provides evidence that adaptive divergence between subpopulations uncoupled from structural variants can be maintained in high gene flow species. Throughout the genome, we have identified loci that are putatively under directional selection and that are therefore of potential importance for adaptation. Hence, our findings have implications for the fisheries management of the European plaice in order to sustain its high adaptive capacity in the North Sea and the Baltic Sea.

## AUTHOR CONTRIBUTIONS

**Peggy Weist:** Formal analysis (lead); visualization (lead); writing – original draft (lead); writing – review and editing (lead). **Sissel Jentoft:** Conceptualization (supporting); methodology (supporting); resources (supporting); supervision (supporting); writing – original draft (supporting). **Ole K. Tørresen:** Formal analysis (supporting); methodology (supporting); resources (supporting); writing – original draft (supporting). **Franziska M. Schade:** Methodology (supporting); resources (supporting); writing – original draft (supporting). **Christophe Pampoulie:** Resources (supporting); writing – original draft (supporting). **Uwe Krumme:** Conceptualization (equal); funding acquisition (equal); methodology (supporting); project administration (equal); resources (supporting); writing – original draft (supporting). **Reinhold Hanel:** Conceptualization (equal); funding acquisition (equal); project administration (equal); supervision (lead); writing – original draft (supporting).

## Supporting information


Appendix S1

Table S1–S11

## Data Availability

All raw sequences have been deposited in the European Nucleotide Archive (ENA) at EMBL‐EBI under the umbrella project with the accession number PRJEB36682 (https://www.ebi.ac.uk/ena/data/view/PRJEB36682). Individual accession numbers for samples are provided in Table S1. The ENA accession numbers to the annotated reference genome assembly and to the transcriptome assembly are ERZ1305001 and ERZ1300624, respectively.
