# Supplementary material for: The role of genomic signatures of directional selection and demographic history in the population structure of a marine teleost with high gene flow
Source: Ecol Evol. 2022 Dec 8;12(12):e9602. doi: 10.1002/ece3.9602 (PMC9731920; doi:10.1002/ece3.9602)
Supplement: Supplementary file 1 — Appendix S1 [file ECE3-12-e9602-s001.docx]

**Supplementary Information**

## The role of genomic signatures of directional selection and demographic history in the population structure of a marine teleost with high gene-flow

Weist P^1,*^, Jentoft S^2^, Tørresen OK^2^, Schade FM^3^, Pampoulie C^4^, Krumme U^3^, Hanel R^1^

^1^Thünen Institute of Fisheries Ecology, Herwigstraße 31, 27572 Bremerhaven, Germany

^2^Centre for Ecological and Evolutionary Synthesis, Department of Biosciences, University of Oslo, Blindernveien 31, 0316 Oslo, Norway

^3^Thünen Institute of Baltic Sea Fisheries, Alter Hafen Süd 2, 18069 Rostock, Germany

^4^Marine and Freshwater Research Institute, Fornubúðir 5, 220 Hafnafjörður, Iceland

## Appendix S1: Supporting Material and Methods

#### Mapping to the genome of the Japanese flounder and variant filtering

Gene and SNP discovery in non-model organisms often lack the availability of a reference genome, thus, many studies rely on alternative approaches to identify informative SNP loci (Everett, Grau and Seeb, 2011; Andrews *et al.*, 2016). The use of a publicly available reference genome from closely related species provides a promising approach to overcome the need of a reference genome assembly (Everett, Grau and Seeb, 2011; Miller *et al.*, 2015; Galla *et al.*, 2019).

To explore the utility of closely related reference genomes for SNP discovery in European plaice, we used the high-quality and chromosome anchored genome of the Japanese flounder (*Paralichthys olivaceus)* (Shao *et al.*, 2017). Raw plaice reads were adapter and quality trimmed using Trim Galore v.0.3.3 (Krueger, 2015) and mapped against the Japanese flounder genome assembly using Minimap2 v2.2 (Li, 2018). Duplicates were marked using Picard tools and indels were locally realigned with GATK v3.7 (McKenna *et al.*, 2010; DePristo *et al.*, 2011). Variants were called with GATK v3.7 and hard filters were applied following GATK’s recommendations (Van der Auwera *et al.*, 2013): FS > 20, MQRankSum < 12.5, ReadPosRankSum < 2, QD < 5, MQ < 40. Variants were initially filtered to include only biallelic SNPs. Variants within spanning deletions were removed using BCFtools v1.1 (Li, 2011). SNPs with a genotype quality score < 20, and read depth (DP) < 5, or DP > 20 were excluded with VCFtools v0.1.13 (Danecek *et al.*, 2011) as well as SNPs within a physical distance of 10 bp. We excluded SNPs displaying a minor allele count < 2 across all populations and SNPs deviating from Hardy-Weinberg-equilibrium with a p-value < 0.0001 using Plink v1.9 (Purcell *et al.*, 2007). Furthermore, variants with a minor-allele frequency < 0.03 and more than 20% missing data per site were removed. Linkage disequilibrium (LD) decay was calculated across each of the 24 Japanese flounder chromosomes. Pairwise *r^2^* values were calculated between all SNPs per scaffold using VCFtools. Decay plots were created by binning the distance between SNPs in increments of 1 kb and averaging the *r^2^* values within each bin (Elgvin *et al.*, 2017). SNPs with pairwise *r^2^* values > 0.1 within sliding windows of 50 kb SNPs were omitted with Plink. We did a pairwise calculation of the genetic differentiation along the chromosome between individuals from ICE/BEL, ICE/BOR and BEL/BOR in sliding windows of 200 kb with steps of 50 kb based upon the plaice reads mapped against the Japanese flounder genome (**4,248 SNPs)**.

## Supporting Results and Discussion

For species of conservation or management concern, limited genomic resources often impede the inclusion of genomic data into management strategies. Here, we show that the use of a conspecific reference genome greatly improves the ability to determine the linkage decay between SNPs and eventually leads to a high-resolution set of genome-wide distributed SNPs suitable for inference of population structure and demographic history and to explore the genomic regions that underlie fitness-related traits.

Aligning the trimmed plaice sequencing reads to the Japanese flounder reference genome resulted in poor mapping rates (Table S8). On average, only 36.13% ± 0.024% of the reads aligned to the genome and only 22.33% ± 0.023% of the reads were properly paired. Thus, the number of mapped reads to was greater using a more fragmented, but conspecific reference genome. Since mappability is negatively correlated with phylogenetic distance (Galla *et al.*, 2019), fewer reads are expected to map to the flounder genome. Furthermore, the interspecific differences in genomic complexity, such as the presence of repeat regions, and the mutational load prevent mapping algorithms from aligning sequencing reads to the reference. In total, 34 mio. biallelic SNPs were identified of which 329,028 SNPs remained after applying GATK’s hard filters and further quality filtering resulted in 11,928 SNPs before linkage pruning. We analyzed linkage decay separately for each of the flounder chromosomes (Figure S3). After linkage pruning **4,248** SNP remained for potential down-stream analysis. Hence, the resolving power of the new SNP set is strikingly lower than for the original dataset. Based on this, the dataset was only used to further confirm our findings on the heterogenetic differentiation on the putative SV on chromosome 19 (Figure S12). Valid SNPs are most successfully detected using conspecific references (Everett, Grau and Seeb, 2011). Using cross-species reference genomes can produce different diversity estimates (e.g. nucleotide diversity and heterozygosity) that correlate with estimates derived from SNPs discovered using a conspecific approach (Galla *et al.*, 2019).

## References

Andrews, K. R. *et al.* (2016) ‘Harnessing the power of RADseq for ecological and evolutionary genomics’, *Nature Reviews Genetics*. Nature Research, 17(2), pp. 81–92. doi: 10.1038/nrg.2015.28.

Van der Auwera, G. A. *et al.* (2013) ‘From fastQ data to high-confidence variant calls: The genome analysis toolkit best practices pipeline’, *Current Protocols in Bioinformatics*, 43, pp. 11.10.1-11.10.33. doi: 10.1002/0471250953.bi1110s43.

Danecek, P. *et al.* (2011) ‘The variant call format and VCFtools’, *Bioinformatics*, 27(15), pp. 2156–2158. doi: 10.1093/bioinformatics/btr330.

DePristo, M. a. *et al.* (2011) ‘A framework for variation discovery and genotyping using next- generation DNA sequencing data’, *Nature Genetics*, 43(5), pp. 491–498. doi: 10.1038/ng.806.A.

Elgvin, T. O. *et al.* (2017) ‘The genomic mosaicism of hybrid speciation’, *Science Advances*, 3(6), p. e1602996. doi: 10.1126/sciadv.1602996.

Everett, M. V., Grau, E. D. and Seeb, J. E. (2011) ‘Short reads and nonmodel species: Exploring the complexities of next-generation sequence assembly and SNP discovery in the absence of a reference genome’, *Molecular Ecology Resources*, 11, pp. 93–108. doi: 10.1111/j.1755-0998.2010.02969.x.

Galla, S. J. *et al.* (2019) ‘Reference genomes from distantly related species can be used for discovery of single nucleotide polymorphisms to inform conservation management’, *Genes*. MDPI, 10(1), p. 9. doi: 10.3390/genes10010009.

Keenan, K. *et al.* (2013) ‘DiveRsity: An R package for the estimation and exploration of population genetics parameters and their associated errors’, *Methods in Ecology and Evolution*, 4(8), pp. 782–788. doi: 10.1111/2041-210X.12067.

Krueger, F. (2015) *Trim Galore! A wrapper tool around Cutadapt and FastQC to consistently apply quality and adapter trimming to FastQ files*. Available at: http://www.bioinformatics.babraham.ac.uk/projects/trim_galore/.

Li, H. (2011) ‘A statistical framework for SNP calling, mutation discovery, association mapping and population genetical parameter estimation from sequencing data’, *Bioinformatics*. 2011/09/08. Oxford University Press, 27(21), pp. 2987–2993. doi: 10.1093/bioinformatics/btr509.

Li, H. (2018) ‘Minimap2: Pairwise alignment for nucleotide sequences’, *Bioinformatics*, 34(18), pp. 3094–3100. doi: 10.1093/bioinformatics/bty191.

McKenna, A. *et al.* (2010) ‘The genome analysis toolkit: A MapReduce framework for analyzing next-generation DNA sequencing data’, *Genome Research*, 20(9), pp. 1297–1303. doi: 10.1101/gr.107524.110.

Miller, J. M. *et al.* (2015) ‘Harnessing cross-species alignment to discover SNPs and generate a draft genome sequence of a bighorn sheep (*Ovis canadensis)*’, *BMC Genomics*, 16(1), p. 397. doi: 10.1186/s12864-015-1618-x.

Purcell, S. *et al.* (2007) ‘PLINK: A tool set for whole-genome association and population-based linkage analyses’, *The American Journal of Human Genetics*, 81(3), pp. 559–575. doi: 10.1086/519795.

Shao, C. *et al.* (2017) ‘The genome and transcriptome of Japanese flounder provide insights into flatfish asymmetry’, *Nature Genetics*, 49(1), pp. 119–124. doi: 10.1038/ng.3732.

Supporting Figures


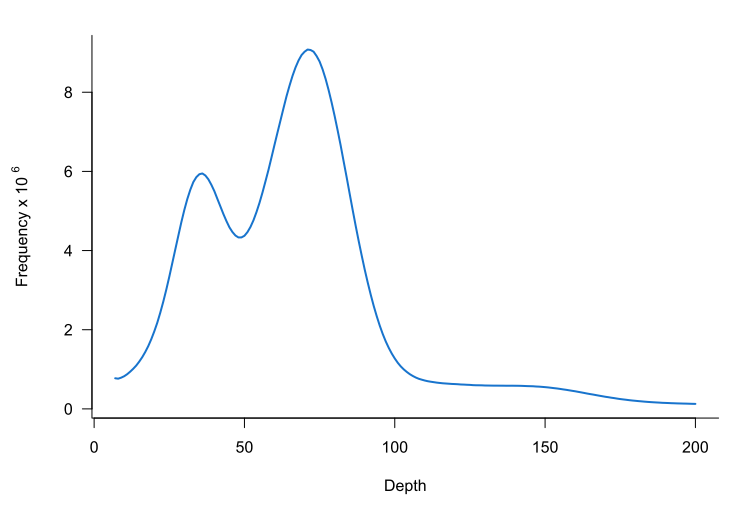


Figure S1. Seventeen-mer estimation of *Pleuronectes platessa* genome size. The genome size, G, was defined by dividing the total number of k-mers by the most frequently occurring frequency. In the present study, K is 17, the total number of k-mers is 41,641,919,389 and depth is 71. Hence, the plaice genome size is estimated to be 587 Mb.

Figure S2. Decay of linkage disequilibrium as a function of distance shown for the twenty longest *P. platessa* scaffolds calculated for the Icelandic (ICE), the Belt Sea (BEL) and the Bornholm (BOR) samples (see Table 1) using the linked dataset (236,458 SNPs).

Figure S3. Decay of linkage disequilibrium as a function of distance shown for each of the *P. olivaceus* chromosomes using the linked dataset (11,928 SNPs).

Figure S4. Decay of linkage disequilibrium as a function of distance shown for plaice scaffolds mapping to the putative structural variant on chromosome 19 of the *P. olivaceus* genome calculated for all plaice samples combined (see table 1) using the linked dataset (236,458 SNPs). Scaffold order corresponds to the relative mapping position on chromosome 19.

Figure S5. Decay of linkage disequilibrium as a function of distance shown for plaice scaffolds mapping to the putative structural variant on chromosome 21 of the *P. olivaceus* genome calculated for all plaice samples combined using the linked dataset (236,458 SNPs). Scaffold order corresponds to the relative mapping position on chromosome 21.


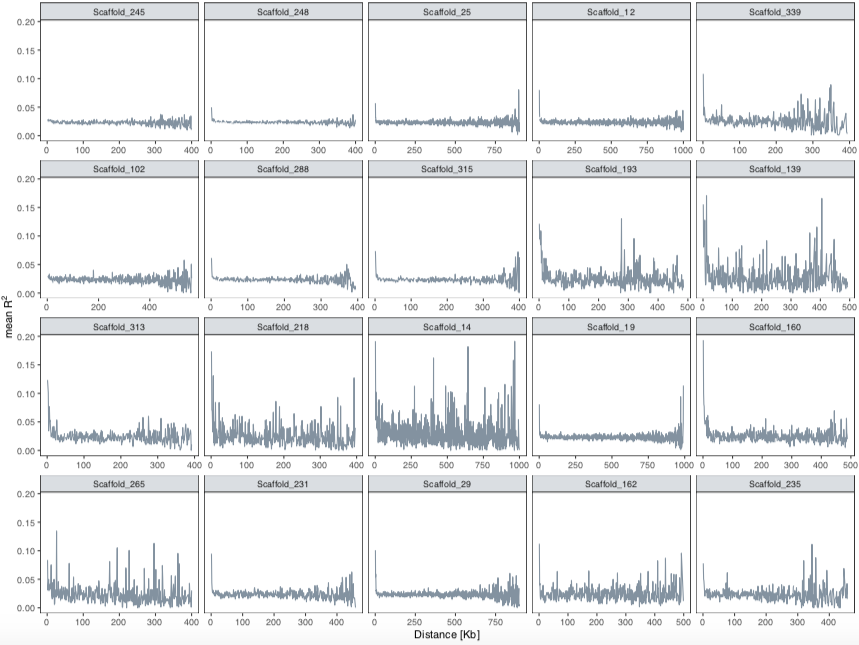


Figure S6. Decay of linkage disequilibrium as a function of distance shown for plaice scaffolds mapping to chromosome 4 of the *P. olivaceus* genome calculated for all plaice samples combined using the linked dataset (236,458 SNPs). Scaffold order corresponds to the relative mapping position on chromosome 4.


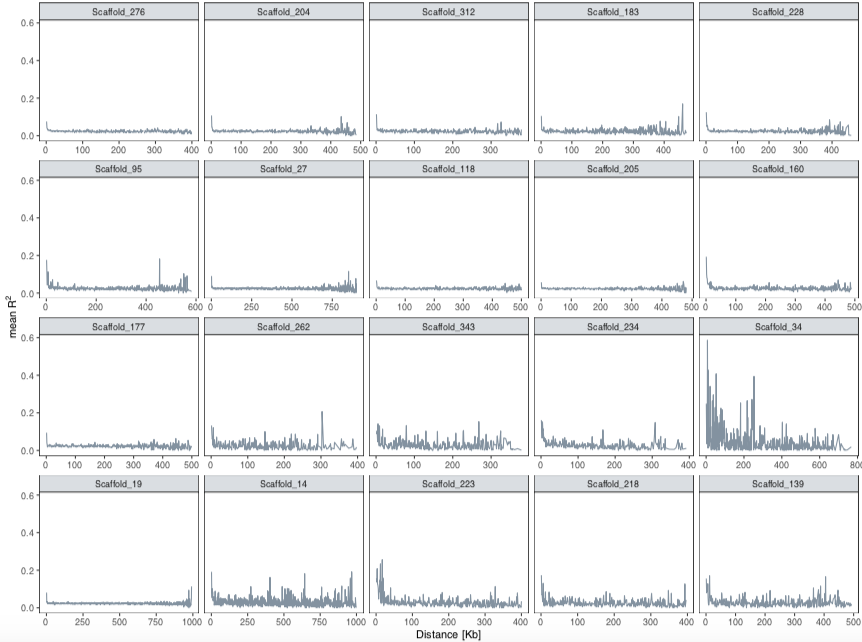


Figure S7. Decay of linkage disequilibrium as a function of distance shown for plaice scaffolds mapping to chromosome 5 of the *P. olivaceus* genome calculated for all plaice samples combined the linked dataset (236,458 SNPs). Scaffold order corresponds to the relative mapping position on chromosome 5.


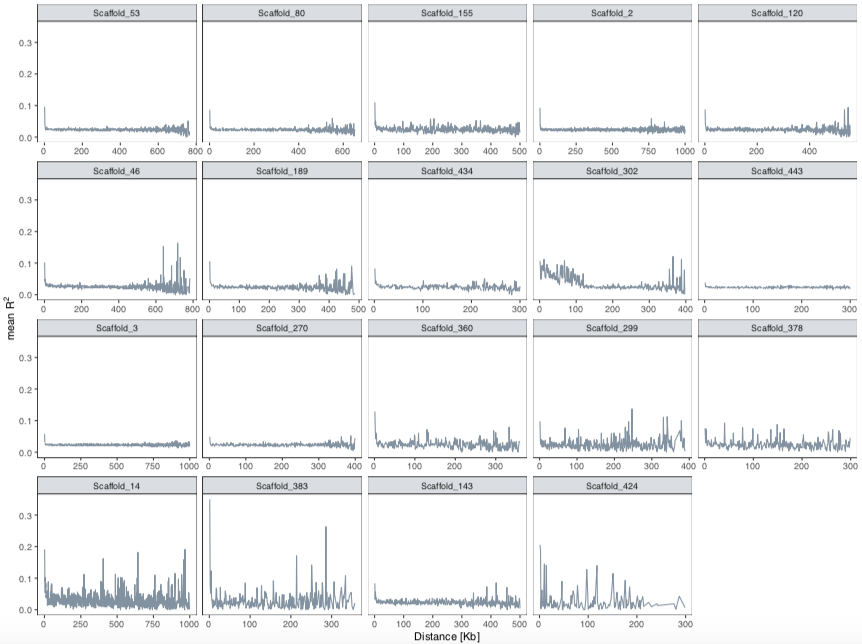


Figure S8. Decay of linkage disequilibrium as a function of distance shown for plaice scaffolds mapping to chromosome 6 of the *P. olivaceus* genome calculated for all plaice samples combined the linked dataset (236,458 SNPs). Scaffold order corresponds to the relative mapping position on chromosome 6.

Figure S9. Investigation of the genetic variation using SNPs from scaffolds showing high linkage decay and mapping to the genome of Japanese flounder through PCA analysis and by plotting the first principal component (PC1) against the individual heterozygosity (Het). (a-d) Plaice scaffolds mapping to chromosome 4. (e-h) Plaice scaffolds mapping to chromosome 5. (i-l) Plaice scaffolds mapping to chromosome 6.

Figure S10. Pairwise calculation of the genetic differentiation along chromosome 19 of the Japanese flounder between individuals from ICE/BOR, ICE/BEL and BEL/BOR in sliding windows of 200 kb with steps of 50 kb based upon the plaice reads mapped against the Japanese flounder genome (**4,248 SNPs)**.

Figure S11. Investigation of the genetic variation using SNPs from scaffolds mapping to chromosome 19 of Japanese flounder through PCA analysis and by plotting the first principal component (PC1) against the individual heterozygosity (Het). (a-d) Plaice scaffolds mapping inside the putative structural variant on chromosome 19 showing high linkage decay. (e-h) Plaice scaffolds mapping inside the putative structural variant on chromosome 19 showing low levels of linkage decay. (i-l) Plaice scaffolds mapping outside the structural variant.


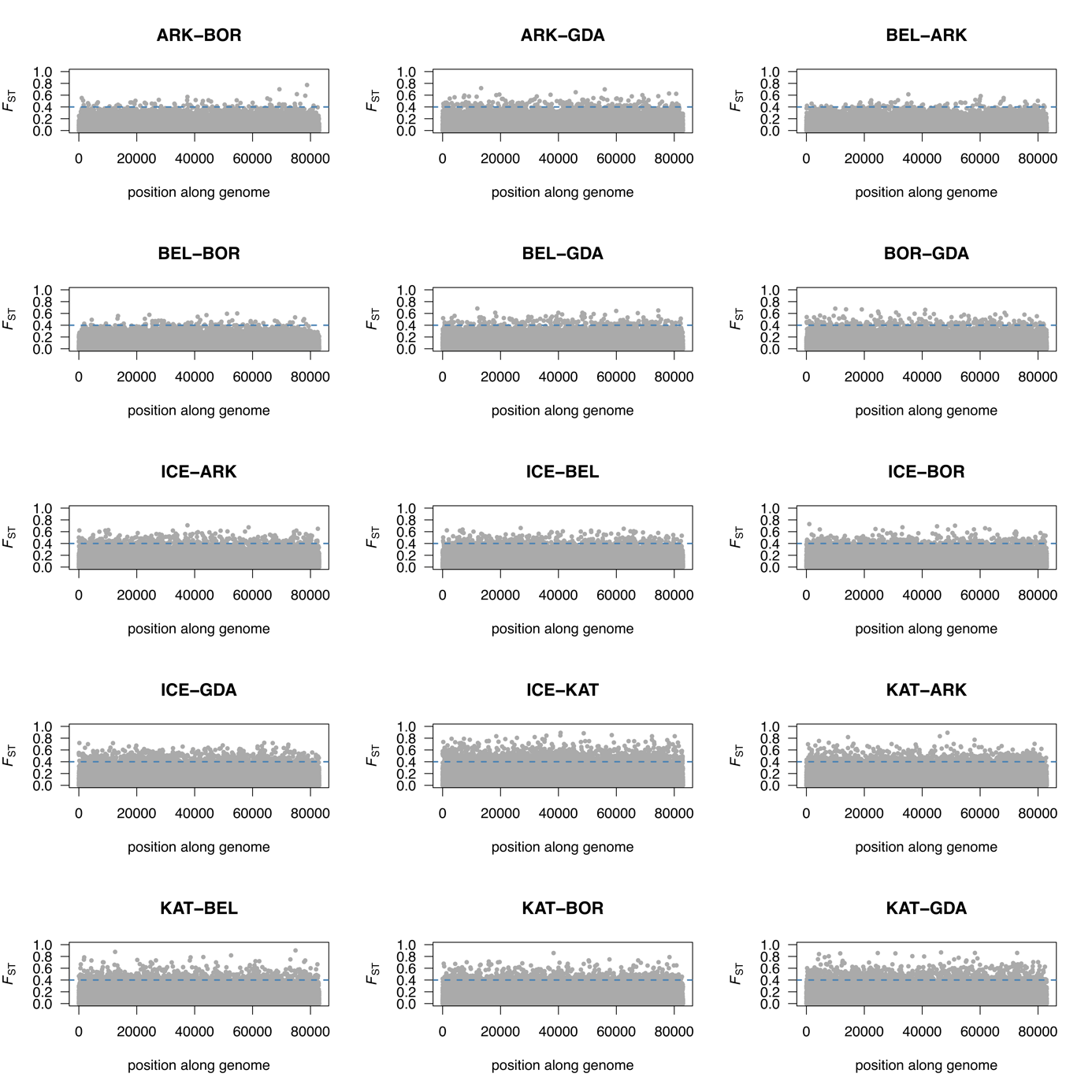


Figure S12. Pairwise differentiation (*F*_ST_) between sampling locations (ICE, KAT, BEL, ARK, BOR, GDA) along the genome (scaffolds were sorted decreasingly by size). The blue dashed line indicates the cutoff used for identifying highly differentiated loci.


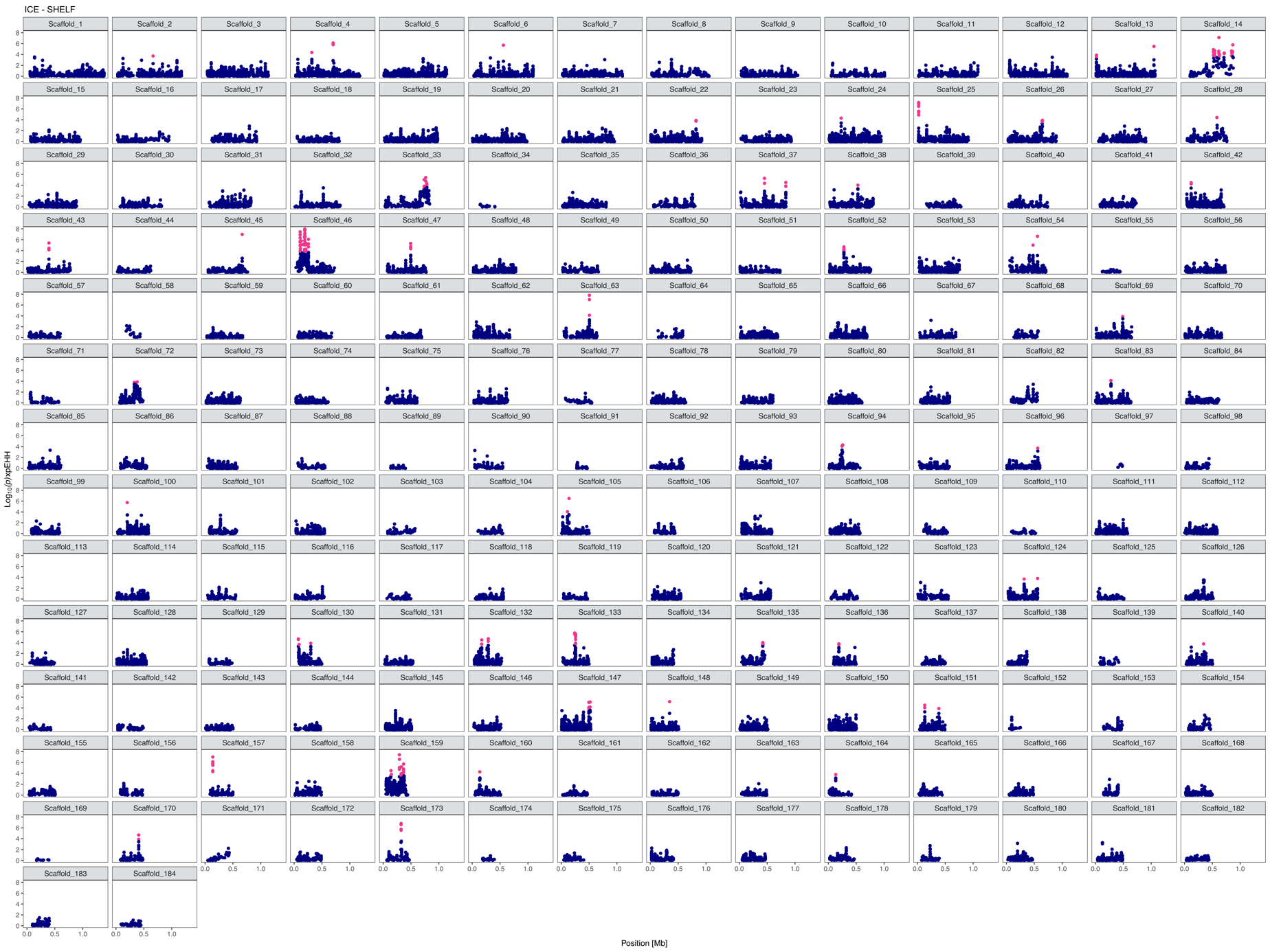


Figure S13. Cross-population extended haplotype homozygosity (xp-EHH) between plaice samples from Iceland and continental shelf samples for scaffolds > 500 kb. Pink dots denote xp-EHH outliers with Log (p-value) above 99.95^th^ percentile.


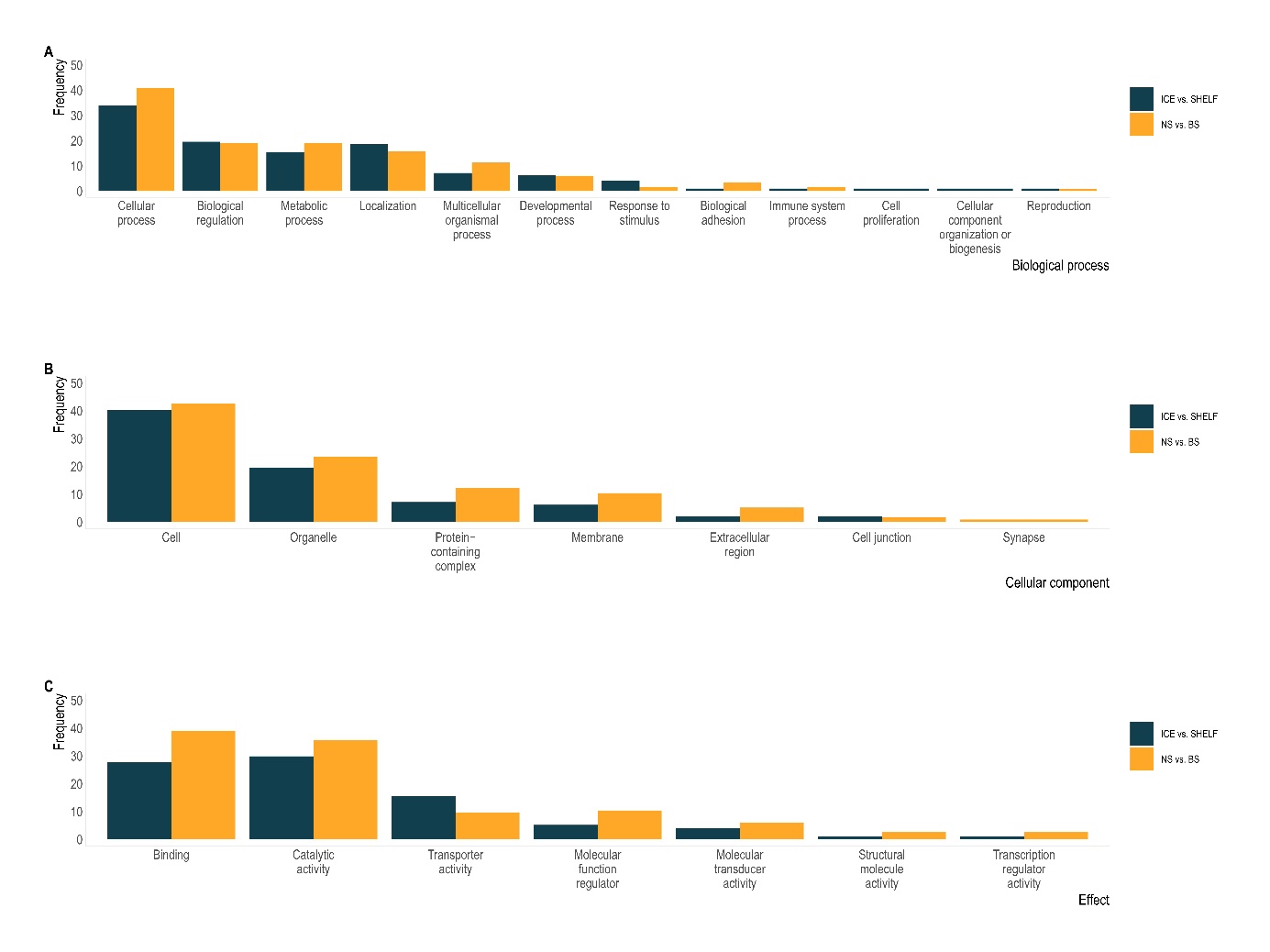


Figure S14. The PANTHER GO classification of candidate genes identified by xp-EHH analyses between the North Sea (NS) and the Baltic Sea (BS) and between Iceland (ICE) and the continental shelf (SHELF) samples (Table 1). Genes were mapped against the human GO-database.

Figure S15. Directional relative migration rates between sampling locations based on 78,508 neutral SNPs. (a) Relative migration rate was calculated based on G_ST_ using the R package diveRsity (Keenan *et al.*, 2013). Each population node is connected to every other node by two connections, representing the two reciprocal gene flow components. The shading and length of the connection are determined by the relative strength of gene flow. Values are coloured according to the connection’s starting point. A maximum of five randomly chosen individuals from each sampling location was used to achieve a balanced sampling scheme. Abbreviations correspond to sampling locations shown in Figure 1a and Table 1. (b) Relative migration rate calculated analogously to (a) but with the Icelandic samples being excluded.

Figure S16. Inference of effective population size over time. Past population history was inferred based on five independent runs of MSMC2 for three randomly chosen individuals from the Icelandic (ICE) population and the Baltic (BS) population. Results are shown for a low mutation rate of µ =2.0×10^-9^ mutations/site/generation and a generation time of three years. The grey bar visualizes the duration of the Last Glacial Maximum (LGM).

Figure S17. Inference of effective population size over time. (a) Past population history was inferred based on five independent runs of MSMC2 for three randomly chosen individuals from the Icelandic (ICE) population and the Baltic (BS) population. Results are shown for a high mutation rate of µ =3.7×10^-8^ mutations/site/generation and a generation time of three years. The grey bar visualizes the duration of the Last Glacial Maximum (LGM).
